# Efficacy and Safety of Domperidone in Combination with Proton Pump Inhibitors in Gastroesophageal Reflux Disease: A Systematic Review and Meta-Analysis of Randomised Controlled Trials

**DOI:** 10.3390/jcm11185268

**Published:** 2022-09-07

**Authors:** Nur Fathurah Zamani, Afifah Sjamun Sjahid, Tuan Hairulnizam Tuan Kamauzaman, Yeong Yeh Lee, Md Asiful Islam

**Affiliations:** 1 Department of Emergency Medicine, School of Medical Sciences, Universiti Sains Malaysia, Kota Bharu 16150, Malaysia; 2 Hospital Universiti Sains Malaysia, Kota Bharu 16150, Malaysia; 3 Department of Medicine, School of Medical Sciences, Universiti Sains Malaysia, Kota Bharu 16150, Malaysia; 4 GI Function & Motility Unit, Hospital Universiti Sains Malaysia, Kota Bharu 16150, Malaysia; 5 Department of Haematology, School of Medical Sciences, Universiti Sains Malaysia, Kota Bharu 16150, Malaysia; 6 Institute of Metabolism and Systems Research, University of Birmingham, Birmingham B15 2TT, UK

**Keywords:** GERD, domperidone, reflux, prokinetic, PPI

## Abstract

The aims of gastroesophageal reflux disease (GERD) treatment are symptom relief and healing of oesophagitis. Besides proton pump inhibitors (PPIs), prokinetic agents are also commonly prescribed to treat GERD. Domperidone, a well-known antiemetic, is an example of a prokinetic agent. It is a dopaminergic blocker that increases lower oesophagus sphincter pressure and activates gastric motility. We carried out a systematic review and meta-analysis to explore the benefits of domperidone in addition to PPI therapy for GERD. We searched for publications comparing PPI plus domperidone to PPI monotherapy in terms of symptom improvement in GERD (until 21 April 2022) on PubMed, Scopus, Google Scholar, Web of Science, Cochrane Library, WHO’s International Clinical Studies Registry Platform, and ClinicalTrials.gov without restricting date, language, or study design. The protocol was registered in PROSPERO (CRD42021242076). This meta-analysis incorporated 11 studies with a total of 841 participants (419 in the PPI plus domperidone group and 422 in the PPI monotherapy group). The combination of a PPI and domperidone resulted in a significant reduction in global GERD symptoms. Adverse events associated with PPI plus domperidone treatment were similar to those associated with PPI monotherapy. In conclusion, the combination of domperidone and a PPI is generally safe and effective in treating GERD as compared with that of PPI alone.

## 1. Introduction

Gastroesophageal reflux disease (GERD) is defined as a condition that develops when the reflux of stomach content causes irritating symptoms and/or complications. GERD is a major public health issue that is becoming increasingly common. It is associated with a significant economic burden and a lower quality of life. Based on the latest meta-analysis, the global prevalence of GERD was estimated as 13.9% and varied substantially across regions (from 12.8% in Latin America and the Caribbean to 19.5% in North America) and nations (from 4.1% in China to 22.4% in Turkey). According to the United Nations 2017 Revision of World Population Prospects, 1.03 billion people have GERD [1]. Ho et al. (1994) determined that the prevalence of GERD symptoms was 1.6% in a multiracial Asian country, Singapore [2]. A 2001 re-survey conducted by a similar author revealed a 10.6% increase in heartburn [3]. According to a similar study, endoscopic oesophagitis increased from 3.9% in 1992 to 9.8% in 2001 in Singapore. Although gastroesophageal reflux is primarily a disorder of the lower oesophageal sphincter (LES), it can be caused by a variety of physiological or pathologic factors. The most common cause is transient relaxation of the lower oesophageal sphincter, which is the brief period of inhibition of the lower oesophageal sphincter tone that occurs independently of swallowing. However, their frequency increases postprandially, causing acid reflux in GERD patients. Other contributing factors include low LES pressure, hiatal hernia, oesophageal obstruction, and delayed gastric emptying [4].

Heartburn (a retrosternal burning pain radiating up towards the throat) and acid regurgitation are the classic symptoms of GERD (the perception of stomach content present in the hypopharynx or mouth). Atypical symptoms may include globus, chest pain, dysphagia, cough, throat symptoms, or belching [5]. GERD-related tissue damage includes oesophagitis, Barrett’s oesophagus, and oesophageal cancer. Reflux can cause oesophageal (heartburn, regurgitation) or extra-oesophageal symptoms. Extra-oesophageal GERD symptoms such as chronic cough, asthma, laryngitis, tooth erosions, and gingivitis are common but less well-known. Extra-oesophageal involvement can occur when classic GERD symptoms are missing [6]. A study conducted by Rajendra and Alahuddin [7] in 2004 found a monthly prevalence of heartburn of 9.7% and weekly symptoms of 6% among Malaysian patients. Endoscopic evidence of oesophagitis is estimated to be present in only 20–40% of symptomatic GERD patients [8].

GERD is diagnosed by the symptoms, endoscopy findings, 24-h oesophageal pH monitoring, and the proton pump inhibitor (PPI) test. There is no gold standard test for GERD. GERD can be diagnosed with a simple, non-invasive, inexpensive questionnaire. There are few questionnaires to evaluate and diagnose GERD, such as the Gastroesophageal Reflux Disease Questionnaire (GERDQ) and Frequent Scale for the Symptoms of Gerd (FSSG) questionnaires. The FSSG consists of 12 questions. Each response is scored as follows for the frequency of each symptom: 0—never; 1—rarely; 2—occasionally; 3—frequently; and 4—always [9]. GERDQ evaluation is conducted by asking patients to rate their symptoms and over-the-counter (OTC) medication use over the past week. It uses a four-graded Likert scale (0–3) to score four positive predictors of GERD (heartburn, regurgitation, sleep disturbance due to reflux symptoms, or OTC medications for reflux symptoms) and a reversed Likert scale (3–0) for two negative predictors (epigastric pain and nausea), giving a total GERD score range of 0–18 [10]. However, the questionnaires are not widely used in clinical settings for various reasons [9,10,11].

The aims of treatment in GERD patients are symptom control and healing of oesophagitis where it occurs. The PPI and histamine-2 (H2) receptor antagonist should be used as the mainstay of GERD treatment for acid suppression, with PPIs being clearly superior to H2-receptor (H2R) antagonists [12]. Prokinetic agents have also been used for the treatment of GERD and dyspepsia over the years. Their mode of action includes improving lower oesophageal sphincter function, oesophageal motility, and gastric emptying [13]. Domperidone is a common antiemetic and is widely used in children, especially in cases of acute gastroenteritis, but there is a significant lack of use in adults. Despite its cardiovascular effects, domperidone is readily accessible in Asian countries, inexpensive, and generally safe. It is a blocker dopaminergic that activates gastric motility, decreases tone, and increases the level of LES pressure [14,15].

There have been reports of serious side effects of domperidone. It has 7% central nervous system (CNS) side effects as compared to 20% for metoclopramide. Domperidone also potentially causes toxicity and heart effects (longer Q–T interval and Q–T prolongation) and may also increase the effective refractory period (blocking potassium channels). Compared to metoclopramide, domperidone has a lower risk of cardiovascular events overall at dosages under 30 mg/day and does not cause QT prolongation, according to a 2018 meta-analysis of the drug’s cardiac safety profile [16]. PPIs are widely used in the treatment of GERD worldwide; however, the beneficial effect of prokinetic agents added to PPI therapy has only been demonstrated in a few studies. Systematic reviews and meta-analyses (SRMAs) conducted previously showed that a combination of prokinetics with PPI treatment is more effective than PPI alone in GERD patients [17]. Additionally, multiple SRMA have recently demonstrated similar results. However, there is no SRMA comparing specifically domperidone plus PPI to PPI alone in GERD. Therefore, the purpose of this current SRMA is to determine the efficacy (improvement of symptoms score, heartburn score, reflux frequency, reflux time, oesophageal pH) and safety (adverse events) of domperidone in combination with proton pump inhibitors in gastroesophageal reflux disease.

## 2. Materials and Methods

This SRMA was carried out in accordance with the PROSPERO-approved protocol (CRD42021242076). The methodology and reporting were developed in accordance with the most recent version of the PRISMA statement [18].

### 2.1. Data Sources and Searches

On 21 April 2021, final searches were conducted without regard for date, language, or study design and identified using electronic databases PubMed, Scopus, Google Scholar, Web of Science, and Cochrane Library. The search strategy is summarised in Table S1. We searched our selected randomised controlled trials (RCT’s) reference list for additional studies that could be included in this SRMA. Additionally, we searched for completed and ongoing trials using the World Health Organization’s (WHO) International Clinical Studies Registry Platform and ClinicalTrials.gov. Duplicate studies were eliminated using the EndNote X8 software (Clarivate Analytics, Philadelphia, PA, USA).

### 2.2. Study Selection

Only RCTs comparing domperidone plus PPI to PPI alone in the treatment of GERD were included. The SRMA’s criteria for inclusion were as follows: (1) RCT; (2) population: adults and children with GERD; (3) intervention: domperidone plus PPI; (4) comparison: PPI alone; (5) outcomes: pain score reduction (GERD score, FSSG score), symptom reduction (heartburn, reflux times, and reflux frequency), and oesophageal pH improvement. We did not limit our publications to the English language only. The study excluded PPI combinations with other prokinetic agents besides domperidone. The titles and abstracts were screened, and two authors independently selected eligible RCTs (N.F.Z. and M.A.I.). We collected full-text versions of relevant articles in order to determine whether they met the inclusion criteria. Disagreements between the authors were ironed out during discussions with A.S.S. and T.H.T.K.

### 2.3. Outcome Measures

Our primary outcome measure was the efficacy of domperidone in combination with a PPI in treating GERD as measured by validated reduction GERD symptoms scales (GERD/FSSG scores/heartburn/reflux) pre- and post-treatment, where patients were followed for a period of two to three months. The second primary outcome was the improvement of oesophageal PH pre- and post-treatment.

Additionally, we examined the prevalence of adverse events such as dizziness, nausea, vomiting, headache, galactorrhoea, and nonspecific adverse events as secondary outcomes for domperidone safety assessment.

### 2.4. Data Extraction and Quality Assessment

Two authors (N.F.Z. and M.A.I.) extracted data independently from the full-text reports and Supplemental Materials. We extracted the following data from each eligible study: study design, study size, study population, GERD diagnosis, intervention details (doses and timing), desired outcomes, including GERD symptoms pre- and post-intervention, and the prevalence of adverse events. We attempted to contact the corresponding authors of trials included in this review that lacked or had insufficient data. Disagreements between the authors were resolved through discussion with A.S.S. and T.H.T.K. Two authors (N.F.Z. and M.A.I.) assessed the quality of the included RCTs independently using the Joanna Briggs Institute’s (JBI) critical appraisal tool. If the total score was ≤49 percent, 50–69 percent, or ≥70 percent, the studies were classified as poor quality (high risk of bias), moderate quality (moderate risk of bias), or high quality (low risk of bias) [19,20].

### 2.5. Data Synthesis and Analysis

We calculated mean differences (MDs) and associated 95% confidence intervals (CIs) for continuous data on pain score reduction (GERD and FSSG Scores), symptom reduction (heartburn, reflux times, and reflux frequency), and improvement in oesophageal pH. On the basis of the comparator drugs, subgroup analyses were conducted. For dichotomous data, we calculated odds ratios (ORs) and associated 95% confidence intervals (CIs) for adverse events. Additionally, we determined the prevalence of adverse events and associated 95% confidence intervals (CIs). We attempted to contact the authors in order to obtain any unreported outcomes of interest, such as MDs or standard deviations. All analyses and plots were created using RevMan version 5.4 and the meta (version 4.15-1) and metafor (version 2.4-0) packages of R (version 3.6.3) in RStudio (version 1.3.1093) [21]. In all meta-analyses, the random-effects model was used, and the *I*^2^ statistic was used to determine trial heterogeneity (*I*^2^ > 75% indicating significant trial heterogeneity). To determine the significance of the heterogeneity test, the Cochran’s Q test was used; a *p*-value of 0.05 indicated significant heterogeneity. We observed the results using a “leave-one-out” method as a sensitivity analysis. If there were at least ten studies, we intended to conduct publication bias analysis by constructing a funnel plot and Egger’s test.

## 3. Results

### 3.1. Search Outcomes

**Figure 1:**
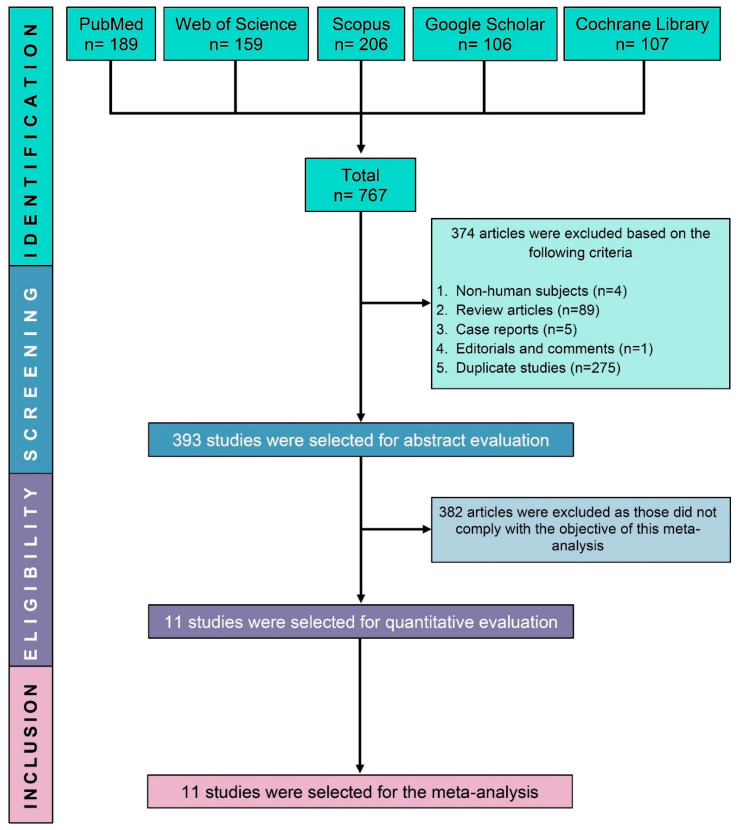
PRISMA flow diagram of study selection.

We identified 767 potentially eligible studies through a search of electronic databases. After excluding 374 non-human, review articles, case reports, and duplicate studies, another 382 studies were excluded based on their abstracts for failing to meet the study’s objective and inclusion criteria. Finally, this SRMA included 11 RCTs (Figure 1).

### 3.2. Study Characteristics

A total of 841 participants were enrolled in the 11 trials, with 419 participants being randomly assigned to receive domperidone plus PPI therapy and 422 participants being randomly assigned to receive PPI alone (either pantoprazole or esomeprazole or omeprazole). The mean age of the participants ranged from 2.45 to 55.45 years old. Six of the included trials were carried out in China [22,23,24,25,26,27], and additional trials were from Indonesia [28], Belarus [29], India [30], Bahrain [31], and Iran [32]. Table 1 summarises the major characteristics of the studies included.

### 3.3. Study Quality

Table S2 provides a detailed quality assessment of the studies that were included. In a nutshell, 81.8% of the included studies were of high quality (low risk of bias), and the remaining two studies were of moderate quality (18.6%). There were no studies of poor quality (high risk of bias) among the studies reviewed.

### 3.4. Primary Outcomes (Efficacy of Domperidone in Combination with PPI)

#### 3.4.1. Modification of Symptom Scores

**Figure 2:**
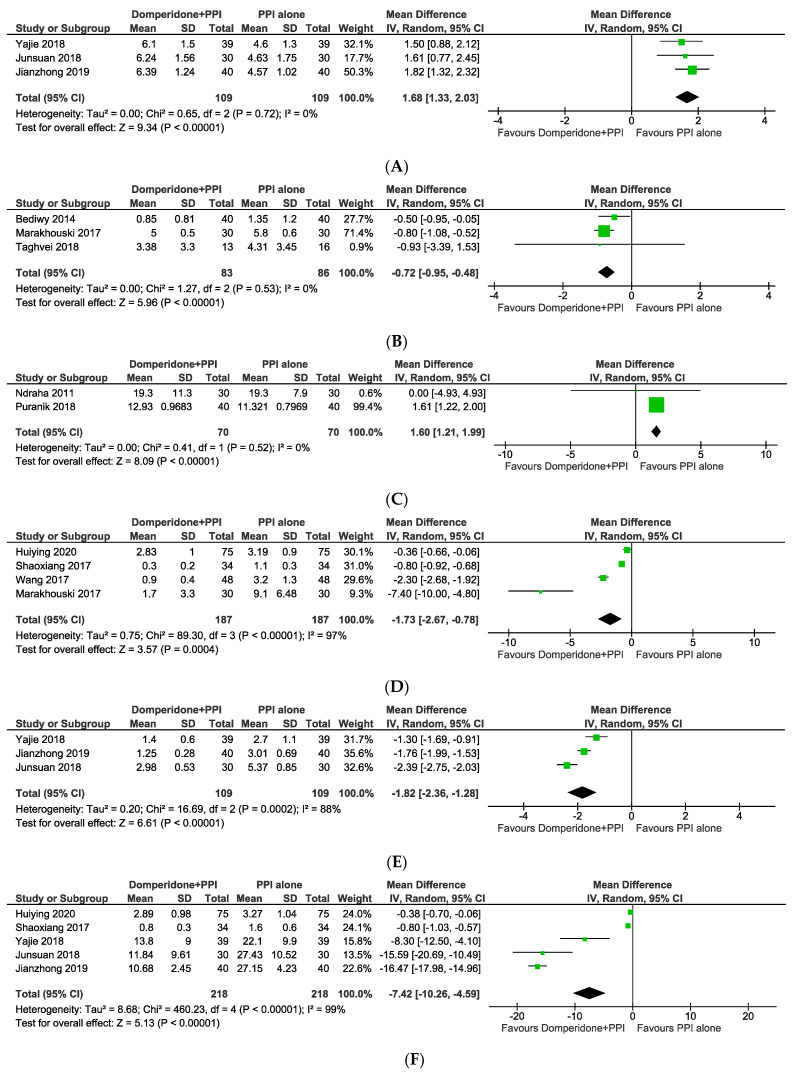
Mean differences of different outcomes including (**A**) oesophageal pH, (**B**) GERD score, (**C**) FSSG score, (**D**) heartburn score, (**E**) reflux time, and (**F**) reflux frequency following a combination of domperidone and proton pump inhibitor vs. proton pump inhibitor alone.

There was a total of five studies that presented detailed symptom scores based on a variety of evaluation scales. FSSG, a GERD-specific questionnaire developed for screening GERD patients, was used in two studies [28,30]. Additionally, the gastroesophageal reflux disease questionnaire was used in three additional studies [29,31,32] (GERD-Q). The GERD-Q and the FSSG were used to measure changes in symptom scores in three trials and two trials, respectively. This study shows that the combined therapy was more effective than monotherapy in the GERD-Q and FSSG subgroups, as shown in Figure 2B,C (MD = −0.72, 95% CI: from −0.95 to −0.48; *p* < 0.00001 and MD = 1.60, 95% CI: from 1.21 to 1.99; *p* < 0.00001).

#### 3.4.2. Heartburn Score, Reflux Times and Frequency

Heartburn score [22,26,27,29] was calculated using a visual analogue scale (VAS) to assess the severity and frequency or the presence of certain symptoms [23,24,25]. Reflux time and frequency were evaluated as the frequency of reflux episodes as well as the length of each individual reflux episode (percent time pH less than 4). There were four studies reporting a heartburn score, five studies reporting reflux times, and three studies reporting a reflux frequency. Figure 2D–F show a statistically significant difference in symptom scores between domperidone-PPI therapy and controls (MD = −1.73, 95% CI: from −2.67 to −0.78, *p* = 0.0004; MD = −1.82, 95% CI: from −2.36 to −1.28; MD = −7.42, 95% CI: from −10.26 to −4.59), all of which were significantly reduced by the combined therapy. However, the heterogeneity in heartburn score (*I*^2^ = 97%, *p* < 0.00001), reflux times (*I*^2^ = 88%, *p* = 0.0002), and reflux frequency (*I*^2^ = 99%, *p* < 0.00001). Combined domperidone and PPI therapy in GERD may be superior to PPI alone in reducing the number of reflux episodes, the duration of acid exposure time, and the heartburn score.

#### 3.4.3. Improvement of Oesophageal pH

Three, five, and three studies reported treatment response in terms of oesophageal pH, symptom score, and symptom improvement, respectively. The oesophageal pH test is a method for measuring the acidity or pH of stomach acid that flows into the oesophagus. Specify by pH below 4. The pooled analysis yielded an MD of 1.68 (95% CI: from 1.33 to 2.03; *p* < 0.00001) with no evidence of heterogeneity (*I*^2^ = 0.0%, *p* = 0.72). (Figure 2A). The results indicated that when domperidone was added to PPI monotherapy, the rate of oesophageal pH responders did not increase significantly.

### 3.5. Secondary Outcomes (Safety of Domperidone in Combination with PPI)

#### Adverse Events

**Figure 3:**
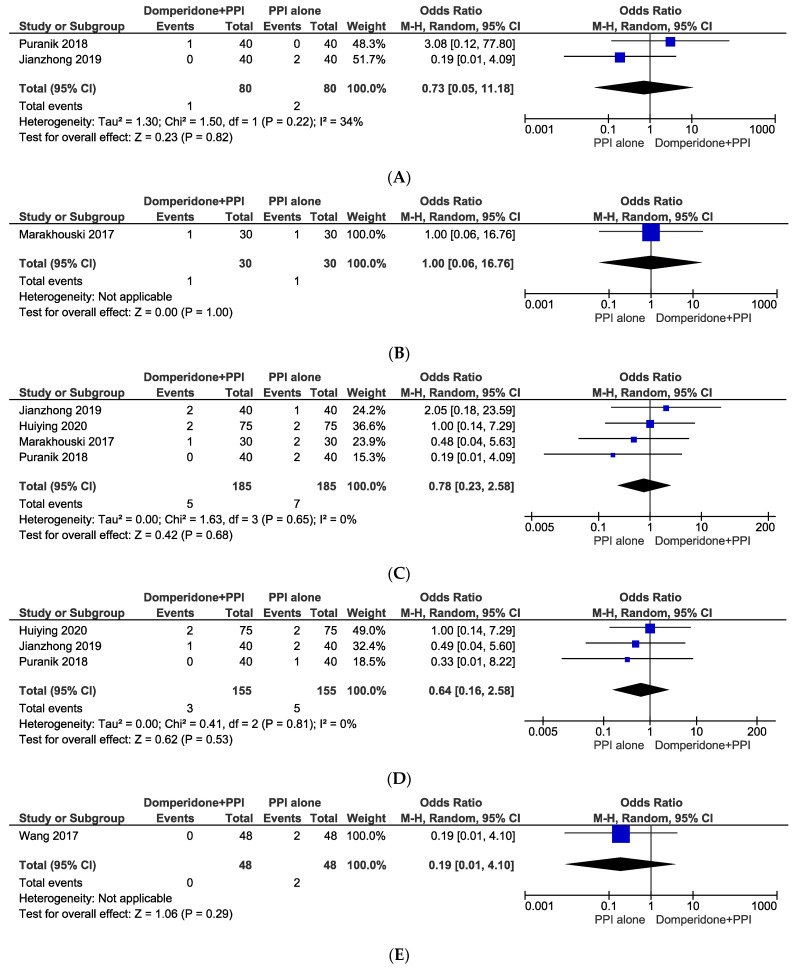
Odds ratios of adverse events including (**A**) diarrhoea, (**B**) galactorrhoea, (**C**) headache, (**D**) nausea and vomiting, and (**E**) weakness and dizziness following a combination of domperidone and proton pump inhibitor vs. proton pump inhibitor alone.

The incidences of diarrhoea, galactorrhoea, headache, nausea with vomiting, and weakness with dizziness were all observed in the included studies as adverse events (Figure 3). In our analyses of the odds of developing adverse outcomes in domperidone in combination with PPI group were diarrhoea (OR: 0.73, 95% CI: from 0.05 to 11.18, *p* = 0.82), galactorrhoea (OR: 1.00, 95% CI: from 0.06 to 16.76, *p* = 1.00), headache (OR: 0.78, 95% CI: from 0.23 to 2.58, *p* = 0.68), nausea and vomiting (OR: 0.64, 95% CI: from 0.16 to 2.58, *p* = 0.53), and weakness and dizziness (OR: 0.19, 95% CI: from 0.01 to 4.10, *p* = 0.29). Due to the lack of statistical significance, it was evident that domperidone in combination with PPI was not more likely to cause adverse effects than the PPI alone group.

### 3.6. Sensitivity Analyses

A sensitivity analysis was conducted by omitting one study at a time to further explore potential sources of heterogeneity. Following the leave-one-out method, none of the results of different outcomes changed except the FSSG score (excluding Puranik 2018); however, there were only two included studies assessing the FSSG score. Due to a lack of studies, it was not appropriate to conduct a subgroup analysis.

## 4. Discussion

GERD is a multifactorial disease with complex pathogenesis. GERD has been considered a continuum of LES dysfunction since the 1960s. Symptomatic but non-erosive GERD is caused by minor dysfunction, such as transient LES relaxations (NERD). Individuals with severe dysphagia develop erosive oesophagitis and/or Barrett’s oesophagus (BE). This continuum represents a pathophysiological decline in LES function, with hiatus hernia and ineffective oesophageal motility serving as contributing factors. Many cases of GERD have predisposing factors and pathophysiology that deviate from this paradigm. Patients with reflux hypersensitivity may have a normal LES but have abnormal sensory processing. Due to the heterogeneity of GERD, clinical management should focus on the pathophysiological characteristics unique to each syndrome. The Montreal Consensus, The Rome Foundation, and the Lyon Consensus all took distinct approaches to resolve this dilemma. Montreal, Rome, and Lyon struggled to identify the “functional” and “physiological” characteristics of the disease [33]. As in our study, Taghvaei et al. enrolled patients with refractory GERD (those who did not respond or partially responded to treatment with pantoprazole (or its equivalent PPI) 40 mg twice a day for one month), whereas the remaining trials included patients diagnosed with GERD but did not describe the specific phenotype of GERD. Even though functional dyspepsia/heartburn and laryngopharyngeal reflux are subtypes of GERD, we did not include them in this study.

Medication therapy for GERD aims to alleviate symptoms and minimise mucosal damage caused by acid reflux [34]. The most potent class of antacid medications are proton pump inhibitors. Although PPIs are first-line medications for GERD, up to one-third of patients do not experience symptom relief after an 8-week course of PPI therapy, and up to 40% of patients with non-erosive reflux disease (NERD) do not respond to PPI therapy once daily in terms of symptom relief [35]. Patients with NERD, erosive gastroesophageal reflux disease, and Barrett’s oesophagus were previously found to benefit from PPI treatment by 60–70%, 20–30%, and 6–10% [36].

In the final step of gastric acid secretion, PPI inhibits the hydrogen-potassium adenosine triphosphatase (i.e., the gastric proton pump). There are numerous PPIs that can be used to treat GERD in children, including omeprazole and esomeprazole, which have been approved for use in >1 year and 1–12 months, respectively. While pantoperazole was found to be well tolerated in infants and children aged from 1 month to 6 years old, it was not well tolerated in those older than 6 years [37,38]. In addition, the 2005 Canadian Consensus Conference [39] and the statements of the World Health Organization Collaborating Centre for Drug Statistics Methodology indicate that all types of PPIs share almost similarities in efficacy [40]. However, PPIs do not reduce the frequency of reflux episodes in adults or children; they only alter the pH of the reflux from acidic to neutral or weakly acidic [41,42]. Due to this variable efficacy, agents other than PPIs are frequently used to treat patients with GERD. These include H2R antagonists, alginate, baclofen, and prokinetics. PPI therapy has been shown to be more effective than H2R agonists and prokinetics in the treatment of GERD, and some meta-analyses have shown that combining prokinetics with PPI is superior to PPI alone. Multiple previous meta-analyses on PPI plus prokinetics in general, such as mosapride, cisapride, acotiamide, revexepride, and domperidone treatment, indicated that prokinetics had a beneficial effect on reducing GERD symptoms [36,43,44].

We demonstrated in this SRMA that the combination of domperidone and PPI therapy was more effective than PPI alone in treating GERD. Endoscopic response, which is ostensibly used to assess treatment effectiveness, is not included in studies, but we do have oesophageal pH monitoring. Analyses of trials involving FSSG and GERD-Q indicate that combined therapy results in greater symptom relief. Additionally, the results indicated that the combination improved heartburn, reflux frequency, and duration more than the PPI alone. Prokinetic agents, approved for gastroparesis [45], have also been suggested as add-on therapy in some refractory GERD patients since delayed gastric emptying can lead to symptom persistence. These drugs improve gastric emptying, LES pressure, and oesophageal clearance. It was also demonstrated by Deepak (2011) and a few other studies that combining PPI and prokinetic agents improves healing rates and provides significant symptom improvement when compared to using either agent alone [8]. However, a recent meta-analysis found that the addition of a prokinetic to a PPI does not improve symptom control, but it does improve quality of life [36]. According to a recent publication, prokinetic therapy is not recommended for otherwise healthy patients with isolated overt regurgitation in paediatric population [46]. There is some evidence of domperidone’s efficacy in children (not witnessed in the neonates’ group), as demonstrated by a significant reduction in the number of reflux episodes (but not the reflux index) when compared to placebo, and there were no serious adverse effects associated with either 0.3 mg/kg three times daily or 0.6 mg/kg three times daily domperidone in children The positive result in studies regarding the population of children, GERD in children/new-borns is likely physiological, self-limiting, and treatable by modifying lifestyle and diet.

In terms of geographical location, studies conducted in both Western and Eastern countries have demonstrated the efficacy of a combination treatment consisting of a PPI and domperidone in the reduction of GERD symptoms. Due to cardiovascular concerns, domperidone is not registered in the United States or Europe; as a result, there are few regional studies available [47]. As well, the majority of studies consist of small sample sizes and originate in Asia. Our meta-analysis included both adults and children, and the combination of domperidone and PPI worked well in both groups. However, more convincing evidence would be required to illustrate that these differences in response are affected by patient-related factors such as race, genetics, environment, and culture. A recent (2020) publication—“ACG Clinical Guideline for the Diagnosis and Management of Gastroesophageal Reflux Disease”—recommended against using any prokinetic agent for GERD treatment unless there is objective evidence of gastroparesis [48]. The recommendation was based on low-grade evidence with a high grade of recommendation strength. In western countries, prokinetic is not recommended owing to the lack of paper, evidence-based research, and reports of adverse events associated with its long-term use. In this report, it was also stated that metoclopramide should not be used alone to treat GERD, but combination therapy was not mentioned. As is well-known, one of the pathophysiologies of GERD is dysfunctional oesophagogastric motility, which can be improved by a prokinetic. The ACG prokinetic recommendation is primarily based on the 2014 meta-analysis by Ren et al. [36] regarding the addition of prokinetic to PPI therapy in GERD. The difference is that the meta-analysis included all types of prokinetics, while we only included domperidone. Additionally, in our study, we have a number of included studies published after 2014 [22,23,24,25,26,27,29,30,32]. The results of the other two recent meta-analyses published on a similar topic favoured the use of prokinetics in conjunction with PPIs for the treatment of GERD [35,49].

Despite the fact that the current meta-analysis represents the most comprehensive review to date on the comparison between PPI plus domperidone and PPI monotherapy in the treatment of patients with GERD, it does have some limitations. Firstly, different types of PPIs agents, domperidone doses, and administration routes were used in the ten trials, with no regard to the dosage being taken into consideration. Secondly, the sample sizes of some studies were relatively small, and the duration of treatment varied from one study to the next. A further concern is that the pooled results may be influenced by chance, given the limited amount of data on NERD versus reflux oesophagitis in the studies included. Various phenotypes of GERD should also be considered when examining the evidence of treatment efficacy. The restriction of drug and treatment variation in certain countries should be considered. Lastly, despite the fact that combined therapy improved symptoms, the majority of GERD questionnaires include gastric symptoms as reflux symptoms, and this may be the reason for the improvement. Despite the aforementioned limitations, this study can be considered to have some significant advantages over other studies in its field.

## 5. Conclusions

In conclusion, patients with GERD, either children or adult age group, responded well to a combination of domperidone and PPIs. There is no statistically significant difference between combined therapy and single therapy in terms of side effects. As a result, the combination of domperidone and a PPI is generally safe and effective in treating gastroesophageal reflux disease.

## Figures and Tables

**Table 1:** Major characteristics of the included studies.

No	Study ID [References]	Country	Type of Participants	Disease Condition	Follow-Up Duration	Treatment Strategies	Domperidone + PPI		PPI Alone	
							Number of Participants (% Female)	Age of the Participants (Mean ± SD/Range) Years	Number of Participants (% Female)	Age of the Participants (Mean ± SD/Range) Years
1	Bediwy 2014 [31]	Bahrain	Children	Asthma with GERD	12 weeks	Domperidone 0.5 mg/kg + Esomeprazole 2 mg/kg for 12 weeks	40 (20) (50%)	8.1 ± 1.3	40 (45%)	7.7 ± 1.2
2	Huiying 2020 [26]	China	Adult	Asthma with GERD	1 month	Domperidone 10 mg + Omeprazole 2 × 20 mg for 1 month	75 (37) (49%)	55.4 ± 4.5	75 (47%)	55.1 ± 4.2
3	Jianzhong 2019 [24]	China	Children	GERD	2 months	Domperidone 10 mg + Omeprazole 20 mg for 1 month	40 (16) (40%)	2.5 ± 0.2	40 (35%)	2.4 ± 0.1
4	Junsuan 2018 [23]	China	Children	GERD	8 weeks	Domperidone 3 × (0.3 mg/kg) + IV Omeprazole (0.5–1 mg/kg) for 8 weeks	30 (12) (40%)	7.2 ± 1.4	30 (50%)	7.6 ± 1.5
5	Marakhouski 2017 [29]	Belarus	Adult	GERD	12 weeks	Domperidone 30 mg + Omeprazole 20 mg for 8 weeks	30 (19) (63%)	47.1 ± 10.8	30 (46%)	45.7 ± 13.0
6	Ndraha 2011 [28]	Indonesia	Adult	GERD	2 weeks	Domperidone 3 × 10 mg + omeprazole 2 × 20 mg for 2 weeks	30 (16) (53%)	44.3 ± 12.2	30 (80%)	40.4 ± 13.9
7	Puranik 2018 [30]	India	Adult	GERD	2 weeks	Domperidone 30 mg + Pantoprazole 40 mg for 2 weeks	40 (12) (30%)	19.0–78.0	40 (30%)	19.0–78.0
8	Shaoxing 2017 [22]	China	Adult	GERD	4 weeks	Domperidone 3 × 10 mg + Omeprazole 20 mg for 4 weeks	34 (14) (41%)	22.4 ± 1.4	34 (35%)	22.1 ± 1.2
9	Taghvei 2019 [32]	Iran	Adult	GERD	1 month	Domperidone 3 × 10 mg + Pantoprazole 2 × 40 mg for 1 month	13 (6) (46.2%)	35.9 ± 9.9	16 (68%)	37.3 ± 9.2
10	Wang 2017 [27]	China	Adult	Superficial gastritis	3 weeks	Domperidone 3 × 10 mg + Omeprazole 2 × 20 mg for 3 weeks	48(27) (56.25%)	42.7 ± 2.6	48 (37%)	42.8 ± 2.7
11	Yajie 2018 [25]	China	Children	GERD	2 months	Domperidone 3 × 10 mg + Omeprazole for 2 months	39 (18) (46%)	7.1 ± 2.2	39 (41%)	6.9 ± 2.0

GERD: gastroesophageal reflux disease, PPI: proton pump inhibitor, IV: intravenous.

## Data Availability

Data are contained within the article or supplementary material.

## Supplementary Materials

The following supporting information can be downloaded at: https://www.mdpi.com/article/10.3390/jcm11185268/s1, Table S1: Search strategies; Table S2: Quality assessment of the included randomised-controlled trials.
