# Supplementary material for: Efficacy and Safety of Domperidone in Combination with Proton Pump Inhibitors in Gastroesophageal Reflux Disease: A Systematic Review and Meta-Analysis of Randomised Controlled Trials
Source: J Clin Med. 2022 Sep 7;11(18):5268. doi: 10.3390/jcm11185268 (PMC9505562; doi:10.3390/jcm11185268)
Supplement: Supplementary file 1 [file jcm-11-05268-s001.zip › jcm-1745577-supplementary.pdf]

Table S1. Search strategies

Databases	Search strategies
**PubMed**	((gastroesophageal[Title/Abstract] OR gastrooesophageal[Title/Abstract] OR gastro-esophageal[Title/Abstract] OR gastro-oesophageal[Title/Abstract] OR GERD[Title/Abstract] OR GER[Title/Abstract] OR GORD[Title/Abstract]) AND (proton pump inhibitor[Title/Abstract] OR proton pump inhibitors[Title/Abstract] OR PPI[Title/Abstract] OR omeprazole[Title/Abstract] OR prilosec[Title/Abstract] OR rabeprazole[Title/Abstract] OR aciphex[Title/Abstract] OR pantoprazole[Title/Abstract] OR protonix[Title/Abstract] OR lansoprazole[Title/Abstract] OR prevacid[Title/Abstract] OR esomeprazole[Title/Abstract] OR nexium[Title/Abstract] OR dexlansoprazole[Title/Abstract] OR dexilant[Title/Abstract] OR zegerid[Title/Abstract])) AND (domperidone[Title/Abstract] OR prokinetic[Title/Abstract])
**Scopus**	TITLE-ABS(gastroesophageal OR gastrooesophageal OR gastro-esophageal OR gastro-oesophageal OR GERD OR GER OR GORD) AND TITLE-ABS(proton pump inhibitor OR proton pump inhibitors OR PPI OR omeprazole OR prilosec OR rabeprazole OR aciphex OR pantoprazole OR protonix OR lansoprazole OR prevacid OR esomeprazole OR nexium OR dexlansoprazole OR dexilant OR zegerid) AND TITLE-ABS(Domperidone OR prokinetic)
**Web of Science**	#1: TI=(gastroesophageal OR gastrooesophageal OR gastro-esophageal OR gastro-oesophageal OR GERD OR GER OR GORD) AND TI=(proton pump inhibitor OR proton pump inhibitors OR PPI OR omeprazole OR prilosec OR rabeprazole OR aciphex OR pantoprazole OR protonix OR lansoprazole OR prevacid OR esomeprazole OR nexium OR dexlansoprazole OR dexilant OR zegerid) AND TI=(Domperidone OR prokinetic)<br>Indexes=SCI-EXPANDED, SSCI, A&HCI, CPCI-S, CPCI-SSH, BKCI-S, BKCI-SSH, ESCI Timespan=All years<br>#2: AB=(gastroesophageal OR gastrooesophageal OR gastro-esophageal OR gastro-oesophageal OR GERD OR GER OR GORD) AND AB=(proton pump inhibitor OR proton pump inhibitors OR PPI OR omeprazole OR prilosec OR rabeprazole OR aciphex OR pantoprazole OR protonix OR lansoprazole OR prevacid OR esomeprazole OR nexium OR dexlansoprazole OR dexilant OR zegerid) AND AB=(Domperidone OR prokinetic)

	Indexes=SCI-EXPANDED, SSCI, A&HCI, CPCI-S, CPCI-SSH, BKCI-S, BKCI-SSH, ESCI Timespan=All years<br>#3: #2 OR #1<br>Indexes=SCI-EXPANDED, SSCI, A&HCI, CPCI-S, CPCI-SSH, BKCI-S, BKCI-SSH, ESCI Timespan=All years
**Cochrane Library**	(gastroesophageal OR gastrooesophageal OR gastro-esophageal OR gastro-oesophageal OR GERD OR GER OR GORD):ti,ab,kw AND (proton pump inhibitor OR proton pump inhibitors OR PPI OR omeprazole OR prilosec OR rabeprazole OR aciphex OR pantoprazole OR protonix OR lansoprazole OR prevacid OR esomeprazole OR nexium OR dexlansoprazole OR dexilant OR zegerid):ti,ab,kw AND (Domperidone OR prokinetic):ti,ab,kw
**Google Scholar**	allintitle: (gastroesophageal OR gastrooesophageal OR gastro-esophageal OR gastro-oesophageal OR GERD OR GER OR GORD) (proton pump inhibitor OR proton pump inhibitors OR PPI OR omeprazole OR prilosec OR rabeprazole OR aciphex OR pantoprazole OR protonix OR lansoprazole OR prevacid OR esomeprazole OR nexium OR dexlansoprazole OR dexilant OR zegerid) (Domperidone OR prokinetic)

Table S2. Quality assessment of the included randomised-controlled trials

No	Study ID	Critical appraisal checklist													Yes (%)
		1	2	3	4	5	6	7	8	9	10	11	12	13	
1	Bediwy 2014	Y	Y	N	Y	U	Y	Y	Y	Y	Y	Y	Y	Y	84.6
2	Huiying 2020	Y	Y	U	Y	U	U	Y	Y	Y	Y	Y	U	Y	69.2
3	Jianzhong 2019	Y	Y	U	Y	N	N	Y	Y	Y	Y	Y	Y	Y	76.9
4	Junsuan 2018	Y	Y	U	Y	N	N	Y	Y	Y	Y	Y	Y	Y	76.9
5	Marakhouski 2017	Y	Y	Y	Y	N	N	Y	Y	Y	Y	Y	Y	Y	84.6
6	Ndraha 2011	Y	Y	Y	Y	Y	Y	Y	Y	Y	Y	Y	Y	Y	100.0
7	Puranik 2018	Y	Y	N	Y	Y	U	Y	Y	Y	Y	Y	Y	Y	84.6
8	Shaoxing 2017	Y	Y	Y	U	U	U	Y	Y	Y	Y	Y	U	U	61.5
9	Taghvei 2018	Y	U	Y	Y	Y	Y	Y	Y	Y	Y	Y	Y	Y	92.3
10	Wang 2017	Y	Y	U	Y	Y	Y	Y	Y	Y	Y	Y	Y	Y	92.3
11	Yajie 2018	Y	U	U	Y	Y	Y	Y	Y	Y	Y	Y	Y	Y	84.6

1. Was true randomization used for assignment of participants to treatment groups? 2. Was allocation to treatment groups concealed? 3. Were treatment groups similar at the baseline? 4. Were participants blind to treatment assignment? 5. Were those delivering treatment blind to treatment assignment? 6. Were outcomes assessors blind to treatment assignment? 7. Were treatment groups treated identically other than the intervention of interest? 8. Was follow up complete and if not, were differences between groups in terms of their follow up adequately described and analyzed? 9. Were participants analyzed in the groups to which they were randomized? 10. Were outcomes measured in the same way for treatment groups? 11. Were outcomes measured in a reliable way? 12. Was appropriate statistical analysis used? 13. Was the trial design appropriate, and any deviations from the standard RCT design (individual randomization, parallel groups) accounted for in the conduct and analysis of the trial? Y: Yes, N: No, U: Unclear, NA: Not applicable
